# Oleoylethanolamide induces eosinophilic airway inflammation in bronchial asthma

**DOI:** 10.1038/s12276-021-00622-x

**Published:** 2021-06-02

**Authors:** Eun-Kyung Kwon, Youngwoo Choi, Il-Hee Yoon, Ha-Kyeong Won, Soyoon Sim, Hee-Ra Lee, Hyoung Su Kim, Young-Min Ye, Yoo Seob Shin, Hae-Sim Park, Ga-Young Ban

**Affiliations:** 1 Department of Pulmonary, Allergy and Critical Care Medicine, Kangdong Sacred Heart Hospital, Hallym University College of Medicine, Seoul, Korea; 2 Department of Allergy and Clinical Immunology, Ajou University School of Medicine, Suwon, Korea; 3 VHS Veterans Medical Research Institute, VHS Medical Center, Seoul, Korea; 4 Department of Internal Medicine, Veterans Health Service Medical Center, Seoul, Korea; 5 MD Healthcare Inc., Seoul, Korea; 6 Department of Internal Medicine, Kangdong Sacred Heart Hospital, Hallym University College of Medicine, Seoul, Korea; 7 Allergy and Clinical Immunology Research Center, Hallym University College of Medicine, Dongtan, Korea

**Keywords:** Biomarkers, Innate immune cells

## Abstract

Asthma is a chronic eosinophilic inflammatory disease with an increasing prevalence worldwide. Endocannabinoids are known to have immunomodulatory biological effects. However, the contribution of oleoylethanolamide (OEA) to airway inflammation remains to be elucidated. To investigate the effect of OEA, the expression of proinflammatory cytokines was measured by RT-qPCR and ELISA in airway epithelial (A549) cells. The numbers of airway inflammatory cells and cytokine levels in bronchoalveolar lavage fluid, airway hyperresponsiveness, and type 2 innate lymphoid cells (ILC2s) were examined in BALB/c mice after 4 days of OEA treatment. Furthermore, eosinophil activation after OEA treatment was evaluated by measuring cellular CD69 levels in eosinophils from human peripheral eosinophils using flow cytometry. OEA induced type 2 inflammatory responses in vitro and in vivo. OEA increased the levels of proinflammatory cytokines, such as IL-6, IL-8, and IL-33, in A549 cells. In addition, it also induced eosinophilic inflammation, the production of IL-4, IL-5, IL-13, and IL-33 in bronchoalveolar lavage fluid, and airway hyperresponsiveness. OEA increased the numbers of IL-5- or IL-13-producing ILC2s in a mouse model. Finally, we confirmed that OEA increased CD69 expression (an eosinophil activation marker) on purified eosinophils from patients with asthma compared to those from healthy controls. OEA may play a role in the pathogenesis of asthma by activating ILC2s and eosinophils.

## Asthma: A physiological cannabinoid promotes inflammation

Oleoylethanolamide (OEA), an endocannabinoid similar to those found in cannabis, occurs naturally in the body, and has now been implicated in promoting symptoms of asthma. OEA activates white blood cells called eosinophils and ILC2 cells, which are both parts of our immune response. Researchers in South Korea, led by Ga-Young Ban at Kangdong Sacred Heart Hospital, Hallym University College of Medicine, Seoul, studied the effect of OEA on respiratory system cells from people with asthma and in mice used to model the condition in humans. OEA-induced inflammatory responses associated with asthma by stimulating the release of signaling molecules called cytokines. However, some other endocannabinoids have anti-inflammatory effects. Studying the effects of all endocannabinoids could yield new insights into asthma, with this study opening new possibilities for treating asthma by interfering with OEA-induced signaling responses.

## Introduction

Asthma is a common chronic respiratory disease that affects 1–18% of the population worldwide^1^. The prevalence of asthma has steadily increased over the past decade from 1.6 to 2.2% in Korea. Along with the increasing prevalence of asthma, asthma-related health care costs and mortality have shown increasing trends^2^.

Endogenously generated cannabinoids known as endocannabinoids, including anandamide, 2-arachidonylglycerol (2-AG), palmitoylethanolamide, and oleoylethanolamide (OEA), are known to have immunomodulatory effects on diverse immune cells^3,4^. The majority of studies investigating the immunomodulatory role of endocannabinoids have reported that anandamide and palmitoylethanolamide exert anti-inflammatory effects, while 2-AG exerts both proinflammatory and anti-inflammatory effects^4^. Endocannabinoids, especially 2-AG, act through cannabinoid 2 receptors and induce type 2 (T2) inflammation in human peripheral eosinophils and mouse asthma models^5–8^.

A previous study reported that high levels of blood OEA correlated with a decline in forced expiratory volume in 1 s (FEV1) in patients with cystic fibrosis^9^. In a recent study of severe asthmatic patients, the levels of serum OEA increased with increasing asthma severity^10^. In addition, we have reported significantly increased levels of OEA in patients with aspirin-exacerbated respiratory disease (AERD)^11^. The increased levels of OEA in asthma can be defensive or a pathological mechanism. To our knowledge, unlike those of other endocannabinoids, the mechanism of OEA in airway inflammation remains to be elucidated.

Hence, this study was performed to investigate the effect of OEA on the airway epithelial cell line A549, in a mouse model, and in human primary eosinophils from asthmatic patients.

## Methods

### Cell line and culture conditions

All in vitro experiments were performed on the human airway epithelial cell line A549 (American Type Culture Collection, Rockville, MD, USA). The cells were cultured in RPMI 1640 (Gibco, Grand Island, NY, USA) supplemented with 10% FBS(Gibco) and 1× antibiotic–antimycotic solution (Gibco) and incubated at 37 °C and 5% CO_2_. A549 cells were seeded in a 96-well plate (1 × 10^4^ cells/well for the MTT assay) or a 12-well plate (1 × 10^5^ cells/well for RT-qPCR and ELISA) and were incubated at 37 °C and 5% CO_2_ without serum overnight. After serum starvation, the cells were treated in the presence or absence of LTE4 (100 nM: Cayman Chemical Company, Ann Arbor, MI, USA) and OEA (100 µM: TOCRIS, Minneapolis, MN, USA) for 24 h or were pretreated with LTE4 (100 nM) for 6 h and subsequently treated with OEA (100 µM) for 18 h. The treatment concentration of OEA was decided after cell viability test with OEA on A549 cells (Fig. S1).

### MTT assay

Thirty microliters of 3-(4, 5-dimethyl thiazol-2-yl)-2, 5-diphenyl tetrazolium bromide (MTT) solution (5 mg/mL) were added to 96-well plates, and the cells were incubated for 2 h. The medium containing MTT was removed, and 50 µL of dimethyl sulfoxide (DMSO; Sigma-Aldrich, St. Louis, MO, USA) was added. The optical density of formazan was measured using an automated spectrophotometric plate reader at 570 nm.

### qRT-PCR

Total RNA was isolated from A549 cells using a PureLink RNA mini kit (Invitrogen, Carlsbad, CA, USA) according to the manufacturer’s instructions. The RNA was reverse transcribed into cDNA with the ReverTraAce qPCR RT Kit (TOOBO, Osaka, Japan). The following primers (Bioneer, Daejeon, South Korea) were used: human IL-1β (forward: 5′-ACC TGA GCT CGC CAG TGA A-3′; reverse: 5′-TCG GAG ATT CGT AGC TGG AT-3′), human IL-6 (forward: 5′- CCA GGA GAA GAT TCC AA-3′; reverse: 5′-TTT CTG CCA GTG CCT CTT TG-3′), human IL-8 (forward: 5′-CAT ACT CCA AAC CTT TCC AC-3′; reverse: 5′-AGC CCT CTT CAA AAA CTT CT-3′), human IL-25 (forward: 5′-CGA CCC AGA TTA GGT GAG GA-3′; reverse: 5′-TCC ATC TTC ACT GGC CCT AC-3′), and human IL-33 (forward: 5′-CCA AAG AAG TTT GCC CCA TG-3′; reverse: 5′-AAG GCA AAG CAC TCC ACA GT-3′). Real-time PCR was conducted with THUNDERBIRD SYBR Green Mix (TOOBO) on an ABI PRISM 7300 Sequence Detection System (Applied Biosystems, Carlsbad, CA, USA). Relative target gene expression was calculated by normalization to GAPDH with the 2^−△△Ct^ method, and the results are presented as fold changes relative to the controls.

### Animals and treatments

Female 6-week-old BALB/c mice (Jackson Laboratory, Bar Harbor, ME, USA) were maintained under specific pathogen-free conditions. All experimental protocols were approved by the Institutional Animal Care and Use Committee of Ajou University (IACUC-2017-0067). To investigate the effect of OEA, the mice were intranasally treated with 100 µg of OEA (OEA-alone group) for 4 days. To compare the proinflammatory effect, 100 ng of leukotriene E4 was intranasally administered for 4 days in the LTE4-alone group. In the LTE4 pretreatment group, 4-day treatment of OEA was performed after pretreatment with LTE4 for 3 days to evaluate the synergistic effect between LTE4 and OEA. Furthermore, to evaluate the impact of OEA on the asthma model, the mice were intraperitoneally sensitized with 50 µg of OVA(Sigma-Aldrich) and 2 mg of alum (Thermo Fisher Scientific, Waltham, CA, USA) on days 0 and 14 and intranasally challenged with 1% OVA from day 28 to day 31. In addition, 10 µg of OEA (TOCRIS) alone was intranasally administered from day 32 to day 35.

### Differential cell count

Bronchoalveolar lavage fluid (BALF) cells were counted with a hemocytometer.

These cells were classified as macrophages, neutrophils, or lymphocytes using Wright-Giemsa staining. The percentages of lymphocytes, eosinophils, neutrophils, and macrophages were determined by counting at least 200 leukocytes in a randomly selected area under a light microscope.

### Enzyme-linked immunosorbent assay (ELISA)

The levels of human IL-1β, IL-6, IL-8, IL-25, and IL-33 in the culture supernatants of A549 cells and mouse IL-4, IL-5, and IL-13 in BALF were measured using commercial ELISA kits according to the manufacturer’s instructions (R&D Systems, Minneapolis, MN, USA).

### Lung histology

Lungs were fixed in 4% formalin, embedded in paraffin, and cut into 5-μm sections.

H&E staining was conducted to investigate immune cell infiltration. Masson’s trichrome staining was performed to examine airway smooth muscle and collagen in the lungs. Tissue sections were evaluated using ImageJ (National Institutes of Health, Bethesda, ME, USA).

### Airway resistance measurement

To measure airway resistance to inhaled methacholine (Sigma-Aldrich), the flexi Vent System (SCIREQ, Montreal, QC, Canada) was used. The mice were connected to a computer-controlled, small-animal ventilator and ventilated with a tidal volume of 10 mL/kg at a frequency of 150 breaths per minute. Aerosol methacholine was administered at increasing concentrations (0–25 mg/mL), and then the peak airway response to the inhaled methacholine was recorded.

### Western blotting

The tissues were homogenized using a BeadBlaster 24 tissue homogenizer (Benchmark, Fullerton, CA, USA) in 0.5 mL of RIPA buffer (50 mM Tris/HCl (pH 7.5), 150 mM NaCl, 1% NP-40, 0.5% sodium deoxycholate, and 0.1% SDS). Each mouse lung tissue lysate sample was centrifuged at 15,000×*g* at 4 °C for 20 min. Following SDS-PAGE and transfer to a PVDF membrane (Bio-Rad, Hercules, CA, USA), the membranes were incubated with the following primary antibodies: monoclonal mouse anti-β-actin (Santa Cruz, Dallas, TX, USA), monoclonal mouse anti- extracellular signal-regulated kinase 1/2 (Erk1/2) (Cell Signaling, Danvers, MA, USA), and monoclonal mouse anti-phospho-extracellular signal-regulated kinase 1/2 (pErk1/2) (Cell Signaling).

### Flow cytometry

To detect type 2 innate lymphoid cells (ILC2s) in the lung, cells were washed with ice-cold fluorescence-activated cell sorter (FACS) buffer (PBS containing 1% bovine serum albumin (BSA) and 1 mM EDTA), fixed in 4% paraformaldehyde, and subsequently stained with the following antibodies for 30 min at RT: anti-lineage marker cocktail (BD Biosciences), anti-CD45 (BioLegend, San Diego, CA, USA), anti-CD25 (eBioscience), anti-CD90.2 (eBioscience), anti-ST2 (eBioscience), anti-IL-5 (eBioscience) and anti-IL-13 (eBioscience). To stain for cellular CD69 in human eosinophils, the cells were stained with FITC-conjugated anti-CD69 (BioLegend) antibodies or isotype controls (BioLegend). The cells were analyzed using a FACS Canto II flow cytometer (BD Biosciences). The data were analyzed by FlowJo software version 10.6.0 (FlowJo).

### Peripheral blood eosinophil isolation from asthmatic patients and healthy controls

Overall, 12 asthmatic patients and 8 healthy controls were recruited from the Department of Pulmonary, Allergy and Critical Care Medicine, Kangdong Sacred Heart Hospital, Hallym University College of Medicine, Seoul, Korea. Asthmatic patients who had been diagnosed by allergy specialists according to the Global Initiative for Asthma (GINA) 2020 guideline were enrolled. Peripheral venous blood samples were collected from subjects whose asthma was in a stable state. Written informed consent forms were obtained from all study subjects, and the study was approved by the Institutional Review Board of Kangdong Sacred Heart Hospital (KANDONG 2018-03-010-004). Blood was collected in BD Vacutainer tubes containing acid citrate dextrose solution (BD). Biosciences, Franklin Lakes, NJ, USA). Red blood cells were eliminated by hypotonic lysis. Eosinophils were separated from the polymorphonuclear cell-rich fraction of peripheral blood by an EasySep™ Human Eosinophil Isolation Kit (STEMCELL, Cambridge, MA, USA) according to the manufacturer’s recommendations.

### Statistical analysis

The data are expressed as the mean ± standard error of the mean of at least three independent experiments. *P* values < .05 were considered statistically significant. Statistical analyses were performed using SPSS software version 20.0 (IBM Corp., Armonk, NY, USA).

GraphPad Prism 8.0 software (GraphPad Inc., San Diego, CA, USA) was used to produce the graphs.

## Results

### OEA increases the expression of proinflammatory cytokines in A549 cells

**Fig. 1:**
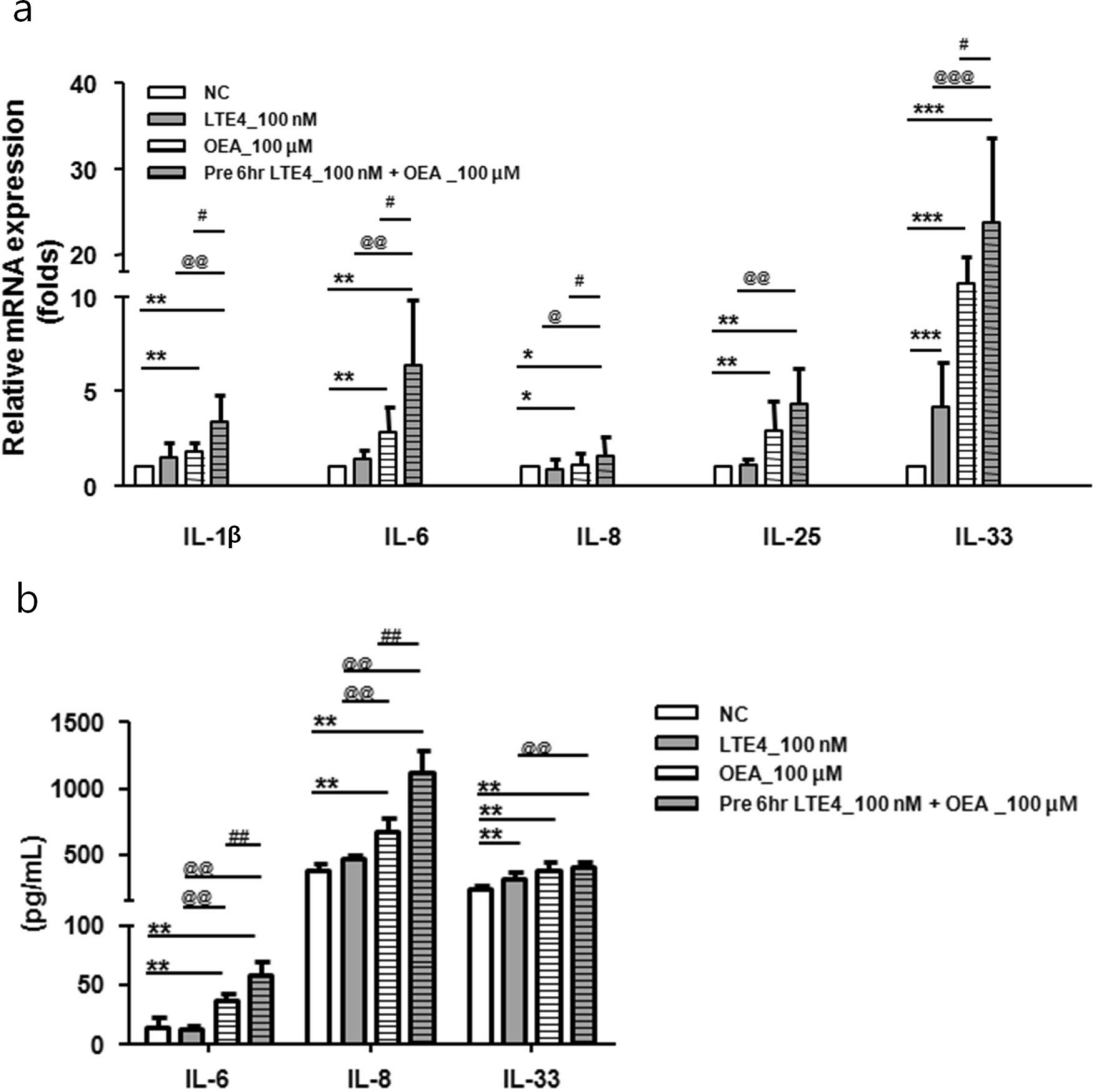
The inflammatory effect of OEA on LTE4-pretreated A549 cells. The cells were treated with LTE4 (100 nM) and OEA (100 μM) for 24 h or were pretreated with LTE4 (100 nM) for 6 h and subsequently treated with OEA (100 μM) for 18 h. **a** The mRNA expression (IL-1β, IL-6, IL-8, IL-25, and IL-33) and **b** production of inflammatory cytokines (IL-6, IL-8, and IL-33) was measured by quantitative real-time PCR and ELISA. The data are presented as fold changes compared with the control group (set at 1) and as the means ± SD of three separate experiments performed in duplicate (*n* = 4–6). *P* values were analyzed by the Mann–Whitney test. ^***^P < 0.001, ^**^P < 0.01, ^*^P < 0.05 vs. NC; ^##^*P* < 0.01, ^#^*P* < 0.05 vs. OEA; ^@@@^*P* < 0.001, ^@@^*P* < 0.01, ^*@*^*P* < 0.05 vs. LTE4.

To investigate the effect of OEA on the expression of proinflammatory cytokines in A549 cells, we measured the mRNA levels of IL-1β, IL-6, IL-8, IL-25, and IL-33. We previously reported that the levels of OEA were elevated in patients with AERD; therefore, the synergistic effect of LTE4 (a major mediator of AERD) was also evaluated^11^. OEA increased all of the mRNA expression in a dose-dependent manner, but LTE4 did not (Fig. S2). In addition, OEA significantly enhanced cytokine production in IL-1β-stimulated A549 cells (Fig. S3). Although the increased mRNA expression of these cytokines was not observed in the LTE4-alone group, the administration of OEA after pretreatment with LTE4 showed a synergistic effect on inflammation in A549 cells. We found that the mRNA expression of IL-1β, IL-6, IL-8, and IL-33 was augmented in the LTE4 pretreatment group compared to the OEA-alone group (Fig. 1a). In addition, OEA markedly increased the protein levels of IL-6, IL-8, and IL-33, as measured by ELISA. The levels of IL-6 and IL-8 were higher in the LTE4 pretreatment group than in the OEA-alone group; however, no significant difference was observed in the level of IL-33 between the groups (Fig. 1b).

### OEA induces inflammatory responses in vivo

**Fig. 2:**
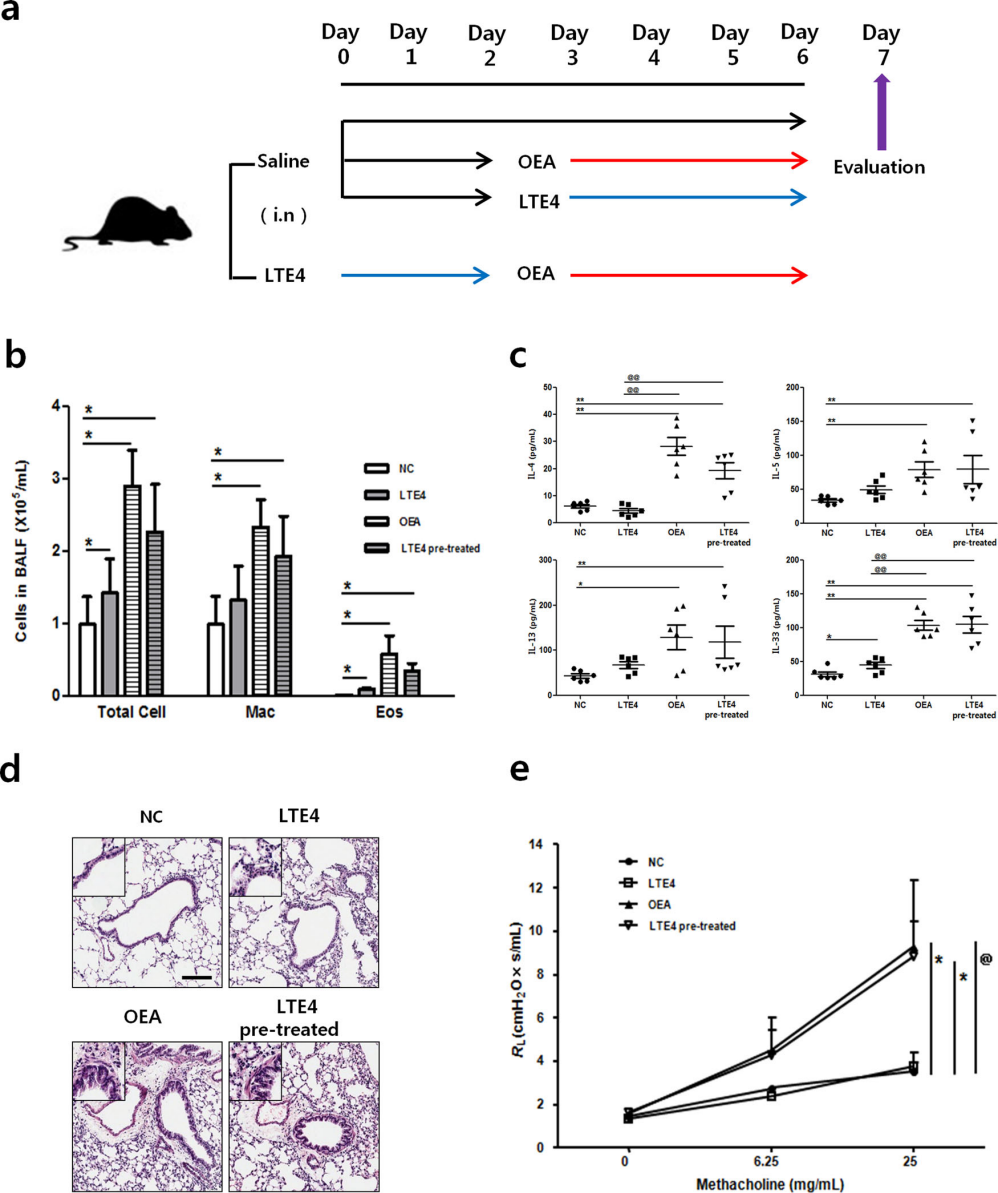
Effect of LTE4 and OEA on pulmonary inflammation in mice. **a** Experimental protocol for LTE4 and OEA treatment. **b** Differential cell counts in BALF. **c** IL-4, IL-5, IL-13, and IL-33 levels in BALF were quantified by ELISA. **d** Lung histology was analyzed by H&E staining. The scale bar indicates 200 μm. **e** Airway hyperresponsiveness. The data are presented as the means ± SD, *n* = 6. *P* values were analyzed by the Mann–Whitney test. ^**^P < 0.01, ^*^P < 0.05 vs. NC; ^@@^*P* < 0.01, ^@^*P* < 0.05 vs. LTE4.

To determine the in vivo effect of OEA, wild-type BALB/c/ mice were intranasally administered OEA-alone and compared to mice in the LTE4-alone and LTE4-pretreated groups (Fig. 2a). The LTE4-alone, OEA-alone, and LTE4-pretreated groups showed significant increases in the numbers of total cells, macrophages, and eosinophils in BALF compared to those in each control group, although there was no significant difference in the number of macrophages between the LTE4-alone and control groups. The LTE4-pretreated group showed no synergistic effect compared to the OEA-alone group (Fig. 2b). Neutrophils and lymphocytes in BALF were not observed in this experiment.

T2 cytokines play important roles during airway remodeling and the development of airway resistance in allergic asthma. To confirm the generation of T2 cytokines in response to OEA, we assessed the cytokine levels in BALF. The levels of T2 cytokines, including IL-4, IL -5, IL-13, and IL-33, in BALF, were higher in the OEA-alone and LTE4-pretreated groups than in the control group; moreover, the levels of IL-4 and IL-33 were higher in the OEA-alone and LTE4 pretreatment groups than in the LTE4-alone group (Fig. 2c).

To identify whether OEA induces lung inflammation, we carried out a histological analysis.

There were typical pathological features of allergic asthma, including inflammatory cell infiltration and basement membrane thickening in both the OEA-alone and LTE4-pretreated groups (Fig. 2d). Consistent with these results, airway hyperresponsiveness (AHR) was analyzed using airway resistance (Rrs) and was significantly increased by 25 mg/mL methacholine treatment in the OEA-alone and LTE4-pretreated groups compared to the control group. However, there was no difference between the OEA-alone and LTE4-pretreated groups (Fig. 2e). Moreover, we administered OEA to a mouse model of OVA-induced asthma to evaluate the impact of OEA on asthma (Fig. S4a). The OEA-alone group showed similar inflammatory responses in the BALF and lung tissues as mice in the OVA-induced asthma model. There was no synergistic effect between OEA and LTE4 in our OVA-induced asthma model (Fig. S4b, c). However, these results indicate that OEA may induce asthmatic inflammation in the mouse lung.

### OEA activates the ERK1/2 signaling pathway in mouse lungs

**Fig. 3:**
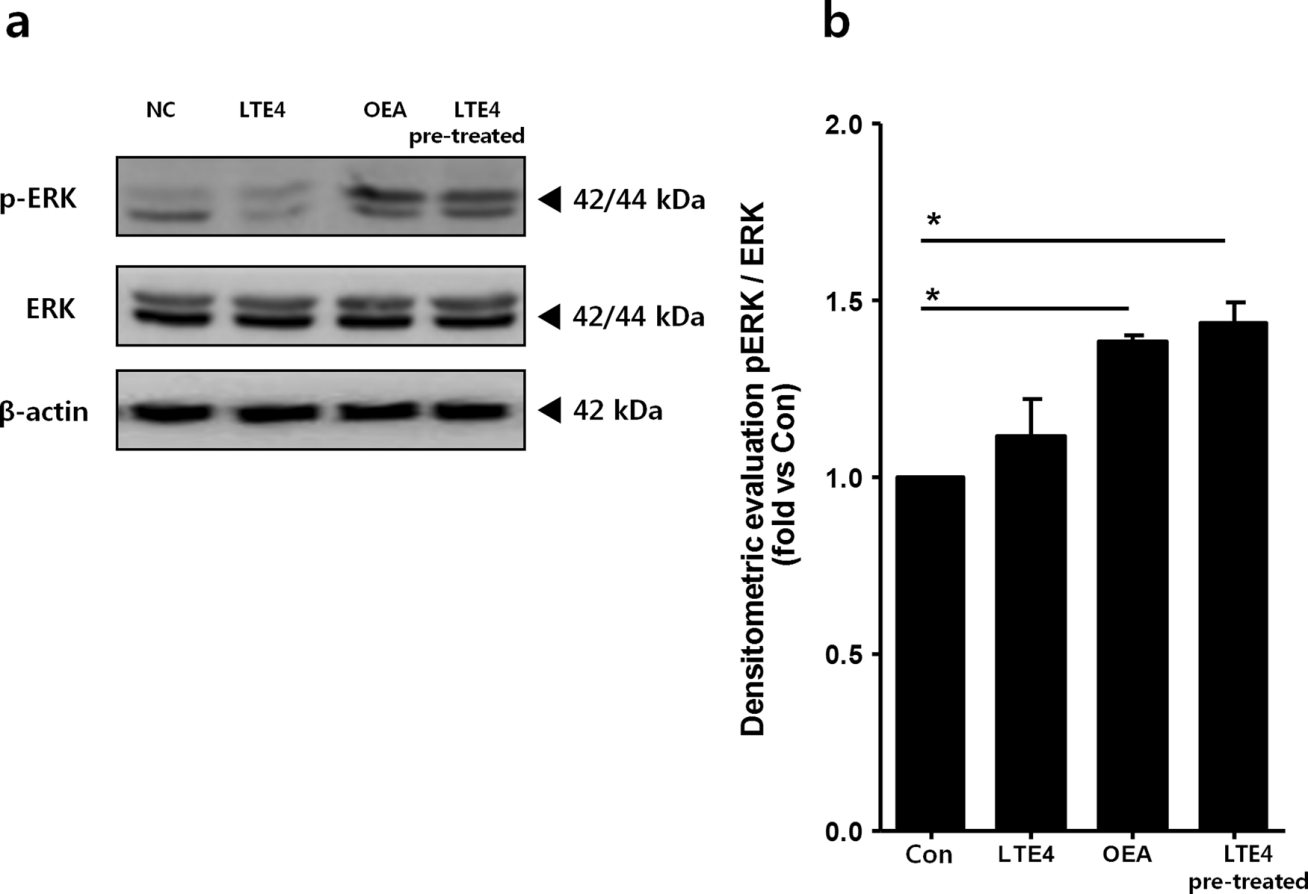
Effect of OEA on ERK activation in mouse lung lysates. **a** The western blot results are representative of three separate experiments. β-actin was used as an internal control. **b** The ratio of phospho-ERK to ERK expression was measured by ImageJ. The data are presented as the mean ± SD, *n* = 3. ^*^P < 0.05; values were analyzed by the Mann–Whitney test compared to the controls. Con control, OEA oleoylethanolamide, LTE4 leukotriene E4, LTE4/OEA pretreated with LTE4 for 6 h and subsequently treated with OEA for 18 h.

ERK1/2 signaling plays an important role in the pathogenesis of asthma. To explore whether OEA regulates intracellular signaling pathways in lung tissue, we examined the activation of the ERK1/2 signaling pathway in mouse lung tissues. The pERK1/2 to ERK1/2 ratio was significantly increased in the OEA-alone and LTE4-pretreated groups compared to the control group, and there were no significant changes in the LTE4-alone group (Fig. 3).

### OEA increases the numbers of IL-5 or IL-13-producing ILC2s infiltrated in the mouse lungs

**Fig. 4:**
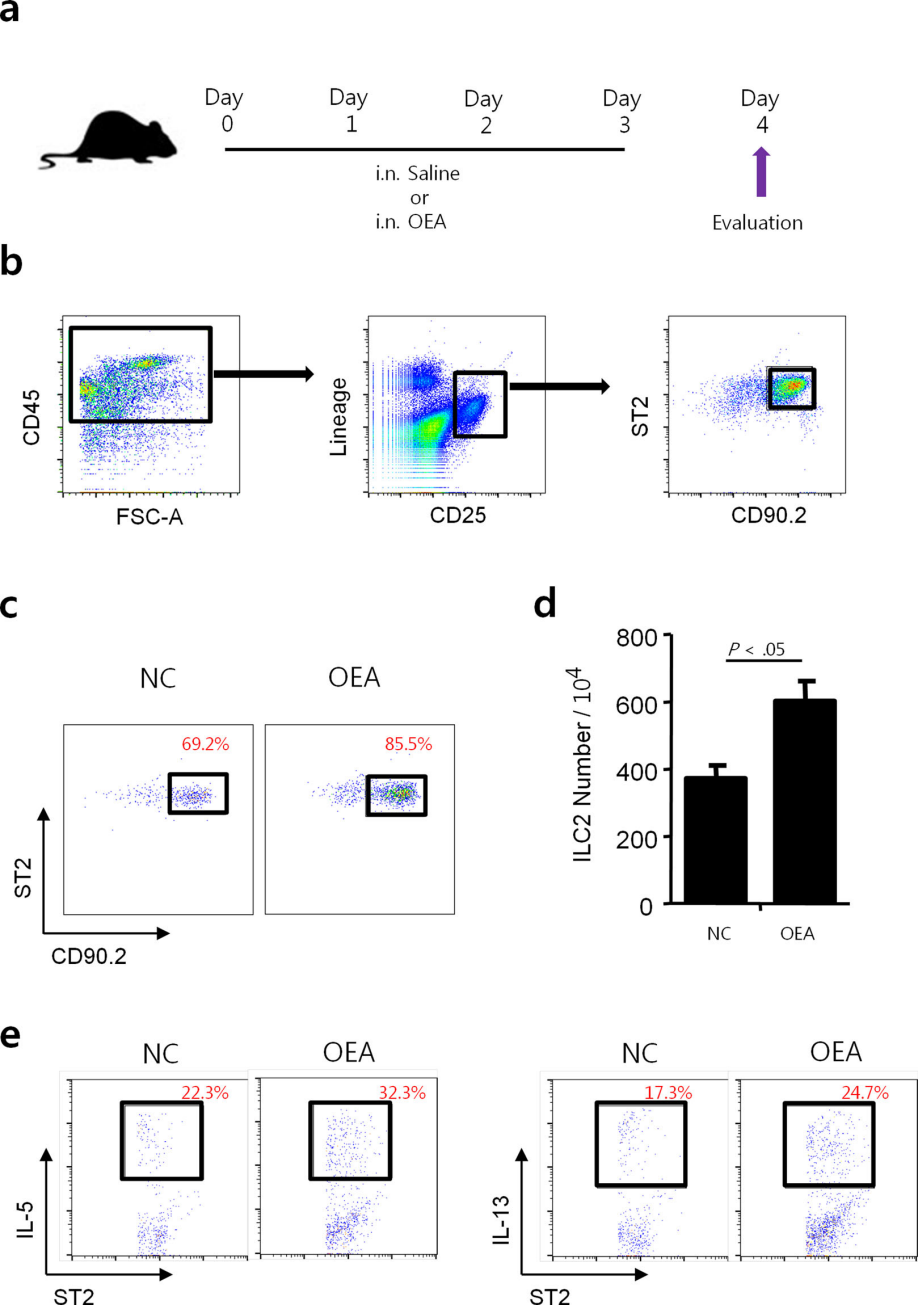
Changes in ILC2 activation in mouse lung tissues after OEA treatment. **a** Schematic of the experimental protocol for OEA treatment. **b** Gating strategy for lung-resident ILC2s. Flow cytometric analysis of ILC2s as defined by a lack of lineage markers and the expression of CD45, CD25, CD90.2, and ST2. **c** ILC2 (%). **d** The number of ILC2s per 104 cells. **e** Quantification of IL-5- or IL-13-producing ILC2s. The data are presented as the mean ± SD, *n* = 3. *P* values were analyzed by the Mann–Whitney test.

ILC2s are crucial mediators of inflammation and tissue remodeling that secrete large amounts of signature cytokines within a short time period^12^. To confirm whether ILC2s are associated with OEA-induced inflammatory responses, mice were intranasally administered OEA for 4 days (Fig. 4a), and lung ILC2s were analyzed using flow cytometry (Fig. 4b).

Interestingly, increased numbers of total ILC2s were detected in the lungs in the OEA-alone group compared to the control group (Fig. 4c, d). In addition, the numbers of IL-5- or IL-13-producing lung ILC2s were elevated when mice were treated with OEA (Fig. 4e). These findings suggest that OEA may be involved in airway inflammation through T2 cytokine-releasing ILC2s.

### OEA enhances the expression of CD69 on peripheral blood eosinophils from asthmatic patients compared to those from healthy controls

**Fig. 5:**
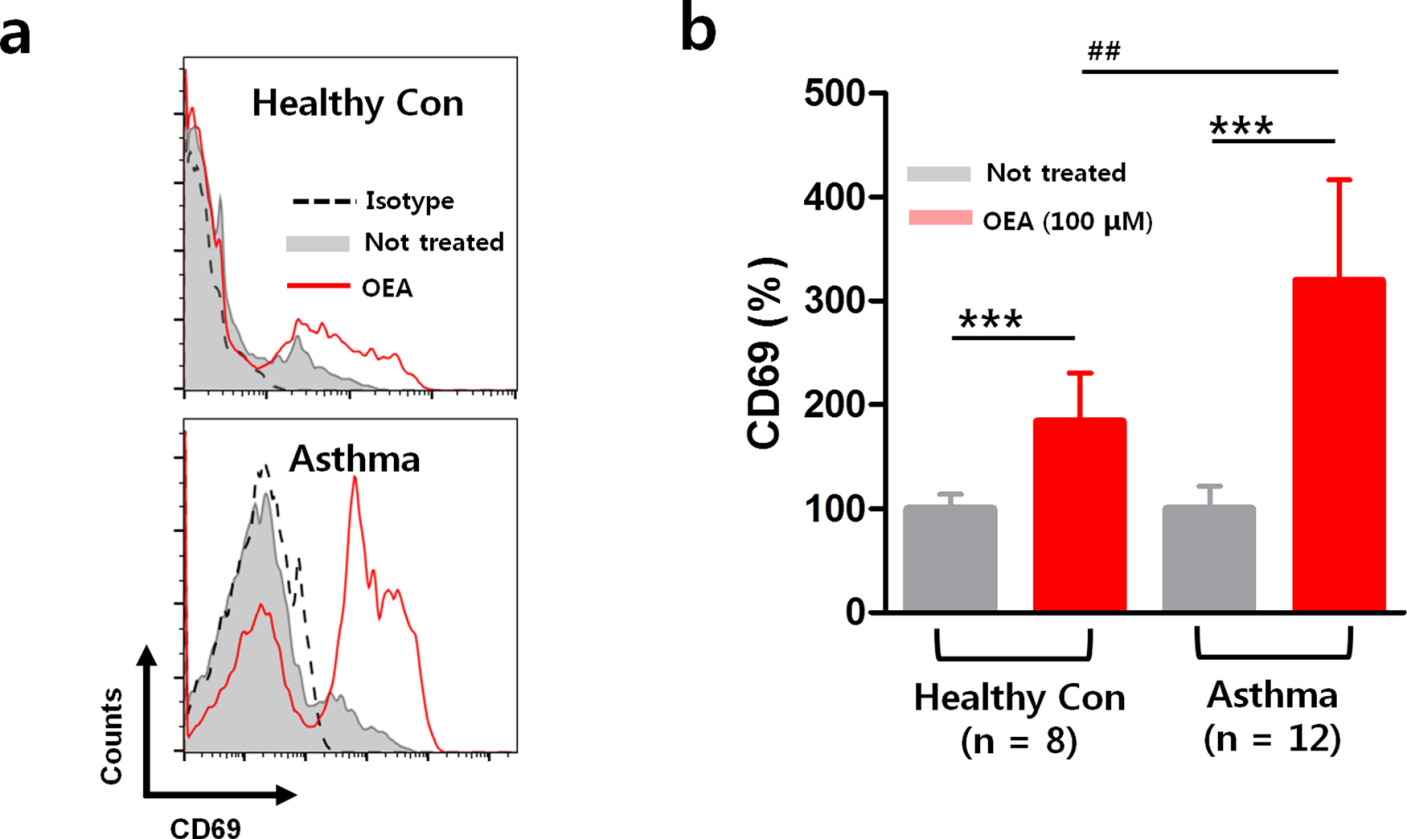
Comparison of eosinophil activation in cells collected from healthy controls and patients with asthma after 24 h of OEA treatment. **a** Representative histogram showing CD69 expression in eosinophils after stimulation with OEA. The fluorescence intensity of eosinophils stained with the isotype control antibody is also shown (broken line). **b** % mean fluorescence intensity of CD69. The data are presented as the means ± SD, *n* = 8, 12. *P* values were analyzed by the Mann–Whitney test. ^***^P < 0.001, ^**^P < 0.01, ^*^P < 0.05 vs. Not treated; ^##^*P* < 0.01, ^#^*P* < 0.05 vs. healthy.

A previous metabolomic study reported that the levels of OEA were elevated in severe asthmatic patients^10^. Demographic data from asthmatic patients are summarized in Table 1. To identify whether OEA impacts eosinophil activation, we measured the expression levels of CD69, a well-known marker of eosinophil activation, in eosinophils isolated from the blood of patients with asthma and healthy controls. The level of CD69 was higher in patients with asthma than in healthy controls (Fig. S5). Interestingly, we observed an increase in CD69 expression in both groups after 24 h of OEA treatment. Flow cytometry showed that the mean fluorescence intensity of CD69 on eosinophils was significantly increased by OEA in patients with asthma compared to healthy controls (Fig. 5a, b).

**Table 1:** Clinical characteristics of the study subjects.

Variables	Asthma (*n* = 12)	HC (*n* = 8)	*P* value
Age (years)	47.9 ± 18.3	36.5 ± 7.3	0.16
Gender (female, %)	5 (41.7%)	4 (50%)	0.79
Atopy (%)	9 (75%)	NA	NA
Severe asthma (%)	4 (33.3%)	NA	NA
FEV1 (% predicted)	84.9 ± 15.5	NA	NA
Peripheral eosinophils (%)	4.2 ± 3.1	NA	NA
Peripheral neutrophils (%)	56.6 ± 7.5	NA	NA
Peripheral eosinophil count (/µL)	367.5 ± 205.5	106.9 ± 65.6	<0.05
Total IgE (IU/mL)	259.4 ± 266.1	NA	NA

*N* number of patients, *HC* healthy control, *FEV1* forced exhaled volume at 1 s, *IgE* immunoglobulin E, *NA* not available.

Values are given as *n* (%) for categorical variables and as mean ± SD for continuous variables. *P* values were analyzed by the Mann–Whitney test.

## Discussion

This is the first study to demonstrate the proinflammatory effect of OEA on asthma models in vitro and in vivo. OEA increased the numbers of T2 cytokine-releasing ILC2s in mice, and these results were consistent with the activation of peripheral eosinophils in asthmatic patients. Therefore, we propose that OEA may contribute to the pathogenesis of eosinophilic inflammation in asthma.

Asthma is a chronic inflammatory disease of the respiratory tract with airway eosinophilia that varies in symptoms and severity over time and exhibits reversible airway obstruction and bronchial hypersensitivity. Various effector cells (mast cells, neutrophils, eosinophils, lymphocytes, and epithelial cells) and cytokines (IL-4, -5, -6, -9, -13, -25, and -33) are known to play important roles in the inflammatory response of asthma. Tissues, cytokines, immune cells, epithelial cells and external factors interact in complex ways, causing inflammation and damage to the airways. This process causes symptoms of asthma such as cough, wheeze, and breathlessness. In addition, it is a complex disease that has heterogeneous phenotypes, ranging from mild to severe, but the underlying disease mechanisms that are associated with disease severity remain poorly understood^13^. A recent metabolomic study reported that 15 metabolites were significantly associated with asthma, and OEA levels increased with asthma severity^10^.

Endocannabinoids are bioactive lipids that activate G-protein-coupled receptors to modulate multiple processes in living organisms, including inflammation. Endocannabinoids include anandamide, palmitoylethanolamide, and OEA^14,15^. OEA, a derivative of the metabolism of oleic acid, is synthesized mainly in small intestine cells, liver and adipose tissues, and neurons. As an endogenous lipid, OEA is bioactive and is involved in feeding control and lipid metabolism^16^. There is no consistency among studies on the role of OEA in inflammation. A recent study showed that OEA treatment decreased the expression of bone marrow dendritic cell surface markers, reduced cell migration, diminished the proliferation of cocultured T cells, and regulated cytokine production in bone marrow dendritic cells, indicating the modulatory effect of OEA on dendritic cell maturation^3^. Moreover, several studies have reported the proinflammatory effect of OEA. OEA was reported to stimulate monocyte migration in a dose-dependent manner and to activate the proinflammatory transient receptor potential vanilloid-type 1 channel^17–19^. Transient receptor potential vanilloid-type 1 channel receptors act as Ca^2+^ ion channels and contribute to chemotaxis, bronchoconstriction, mucous secretion, and airway irritation in asthma pathogenesis^20^. Consistent with these previous reports, we found that OEA treatment of A549 cells enhanced proinflammatory cytokines mRNA and protein expression in an in vitro model. As representative airway epithelial cells, A549 cells have been implicated in the pathogenesis of inflammatory lung disease, such as asthma. Airway epithelial cells are potent sources of proinflammatory substances such as IL-1β, IL-6, and IL-8. IL-6 and IL-8 can enhance the adhesion of leukocytes to airway epithelial cells in asthmatic inflammatory conditions and stimulate cell signaling cascades, resulting in specific immune and inflammatory responses^21^. We have previously reported that OEA levels were elevated in patients with AERD compared with those with aspirin-tolerant asthma and were significantly increased after the lysine-aspirin bronchoprovocation test^11^. Therefore, we examined the synergistic proinflammatory effect of LTE4 (one of the major mediators of AERD) with OEA on A549 cells. In summary, OEA could induce asthma-related cytokines that were augmented by LTE4 in airway epithelial cells.

This study demonstrated that OEA induced inflammatory responses in vivo and in vitro. The numbers of inflammatory cells and the levels of T2 cytokines (IL-4, IL-5, IL-13, and IL-33) in BALF were increased in the LTE4-alone, OEA-alone, and LTE4-pretreated groups, and these factors play important roles in airway inflammation and asthma remodeling. In addition, typical features of asthma, including inflammatory cell infiltration in the lungs and increased airway hyperresponsiveness, were observed in the OEA-alone and LTE4-pretreated groups. However, there were no significant differences between the OEA-alone and LTE4-pretreated groups in vivo. Considering the half-life of LTE4 and the treatment protocol of our experiment, LTE4 may not have synergistic effects with OEA^22^. Further in vivo studies that mimic AERD are needed to confirm the synergistic effect between LTE4 and OEA. In addition, OEA activated ERK1/2 signaling. The ERK1/2 pathway is important for eosinophil priming, degranulation, and cytokine/leukotriene production^23^. The phosphorylation of ERK1/2 was increased in airway biopsy samples from allergic asthmatic patients compared to those of healthy controls^24^. In addition, a single stimulation of cells with cytokines (IL-4, IL-5, and IL-13) causes rapid ERK1/2 activation^25^. Therefore, these results suggest that OEA can elicit histopathological properties of asthma in mouse lungs.

Recently, it has been revealed that ILC2s are present in various tissues and play an important role in mediating damage and the inflammatory response through excessive activation by stimuli. Unlike T cells, which are responsible for the adaptive immune response, ILC2s do not have antigen specificity. IL-25 and IL-33 produced by airway epithelial cells are major cytokines that activate ILC2s that produce T2 cytokines such as IL-5 and IL-13, ultimately leading to increased eosinophils^26–28^. In the present study, we confirmed that OEA increased the levels of IL-33 in BALF, as well the numbers of IL-5- or IL-13-producing ILC2s that infiltrated the lung. Therefore, these findings lead us to hypothesize that OEA can trigger inflammatory reactions in the airway by activating ILC2s.

Although asthma is recognized as having diverse phenotypes and endotypes, eosinophils are one of the most cardinal effector cells in asthma pathogenesis. Eosinophils release a large number of mediators, including basic proteins, cytokines, chemokines, and lipid mediators, which induce airway inflammation, tissue damage, and remodeling. The high degree of eosinophilic inflammation is reported to be closely associated with asthma severity and asthma exacerbation^11,29^. In this study, to evaluate the direct effect of OEA on eosinophil activation in asthmatic patients, peripheral eosinophils were isolated from asthmatic patients and healthy controls and treated with OEA, and CD69 expression on eosinophils was compared between the two groups. CD69 is the most well-known eosinophil surface protein and is involved in eosinophil activation^30–33^. We observed that eosinophils from asthmatic patients were more activated by OEA than those from a healthy control. These finding collectively suggest that OEA is a bioactive lipid that activates eosinophils, which may play a role in asthma pathogenesis.

This study has two limitations. One is that we did not show ILC2 activation by OEA in patients with asthma or healthy controls. The other is that the exact signaling pathway of OEA was not investigated in the present study. Further studies with a number of asthmatic patients according to specific phenotypes/endotypes are warranted to find the signaling axis that is associated with OEA-cognate receptors, and studies to validate our hypothesis are needed.

In summary, OEA may play a role in the pathogenesis of asthma by activating ILC2s and eosinophils. The results of this study may provide novel insights regarding a potential target for asthma management.

## Supplementary information

supporting information
