# Supplementary material for: Oleoylethanolamide induces eosinophilic airway inflammation in bronchial asthma
Source: Exp Mol Med. 2021 Jun 2;53(6):1036–45. doi: 10.1038/s12276-021-00622-x (PMC8257664; doi:10.1038/s12276-021-00622-x)
Supplement: Supplementary file 1 — supporting information [file 12276_2021_622_MOESM1_ESM.doc]

**Supplementary figure legends**

**Figure S1. Effect of OEA on cell viability.** Cell viability (percentage to the control) of A549 cells following 24-hour treatment with different doses of OEA (10-400 μM).

Cytotoxicity was shown at 400 μM OEA, but was not detected at other concentrations. Data are presented as mean ± SD of 3 independent experiments

**Figure S2. OEA induces mRNA expression of inflammatory cytokines on A549 cells.** For the analysis of mRNA expression, cells (1x10^5^) were seeds on a 12-well plate, then treated with variable dose of LTE4 or OEA for 24 hours. The mRNA expressions of IL-1β, IL-6, IL-8 and IL-33 were detected by quantitative real-time PCR. *P* values were analyzed by the Mann Whitney test. Data are presented as mean ± SD of 3 independent experiments. ^***^*P* < 0.001, ^**^*P* < 0.01, ^*^*P* < 0.05; ^##^*P* < 0.01, ^#^*P* < 0.05 between the indicated groups

**Figure S3. Pro-inflammatory IL-1β enhances the inflammatory-induced effect of OEA.** The cells were treated with IL-1β (0.5 ng/mL) and OEA (200 μM) for 24 hours. Inflammatory cytokines for mRNA expression were detected by quantitative real-time PCR. *P* values were analyzed by the Mann Whitney test. Data are presented as mean ± SD of 3 independent experiments. ^***^*P* < 0.001, ^**^*P* < 0.01 and ^*^*P* < 0.05; ^$$^ *P* < 0.01, ^$^*P* < 0.05; ^##^*P* < 0.01 between the indicated groups

**Figure S4. Effect of OEA on OVA-sensitized and challenged mice.**

(**a**) Experimental protocol of OEA treatment. (**b**) Differential cell counts in BALF. (**c**) Representative fields of H&E-stained lungs from mice from the indicated experimental groups. Scale bar, 100 μm. *P* values were analyzed by the Mann Whitney test. Data are presented as mean ± SD of 3 independent experiments. ^**^*P* < 0.01 and ^*^*P* < 0.05 between the indicated groups

**Figure S5. Comparison of activation of eosinophils from patients with and without asthma.**

(**a**) A representative histogram of CD69 expression on eosinophils from patients with and without asthma. (**b**) % mean fluorescence intensity of CD69. Data are represented as means ± SD, n = 8, 12. (**c**) % Relative increased the surface expression of CD69 on eosinophils after treatment of OEA. Data are represented as means ± SD, n = 8, 6, 6. ^**^*P* < 0.01; ^#^*P* < 0.05 between the indicated groups.

***Figure S1***

***
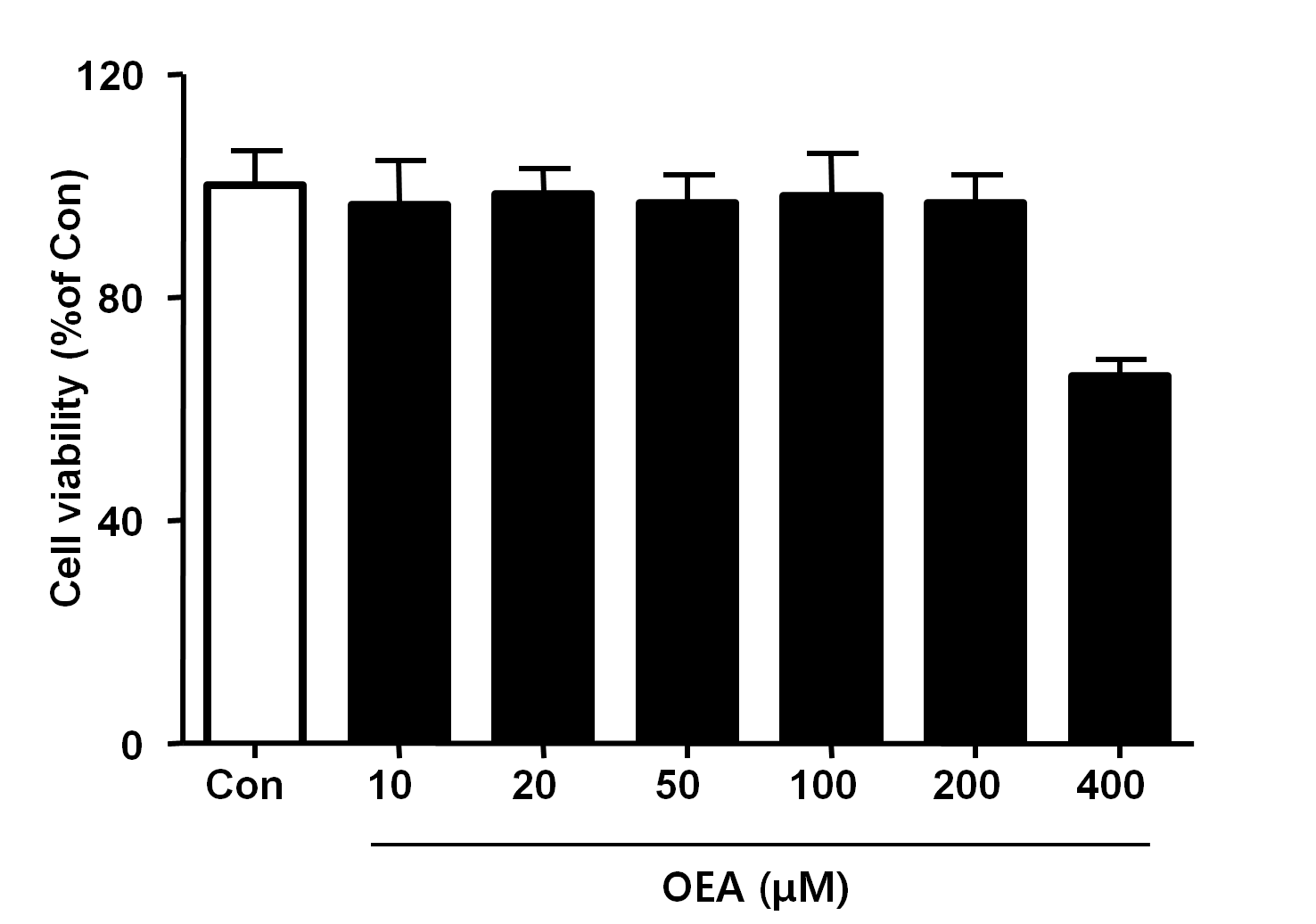
***

***Figure S2***

***
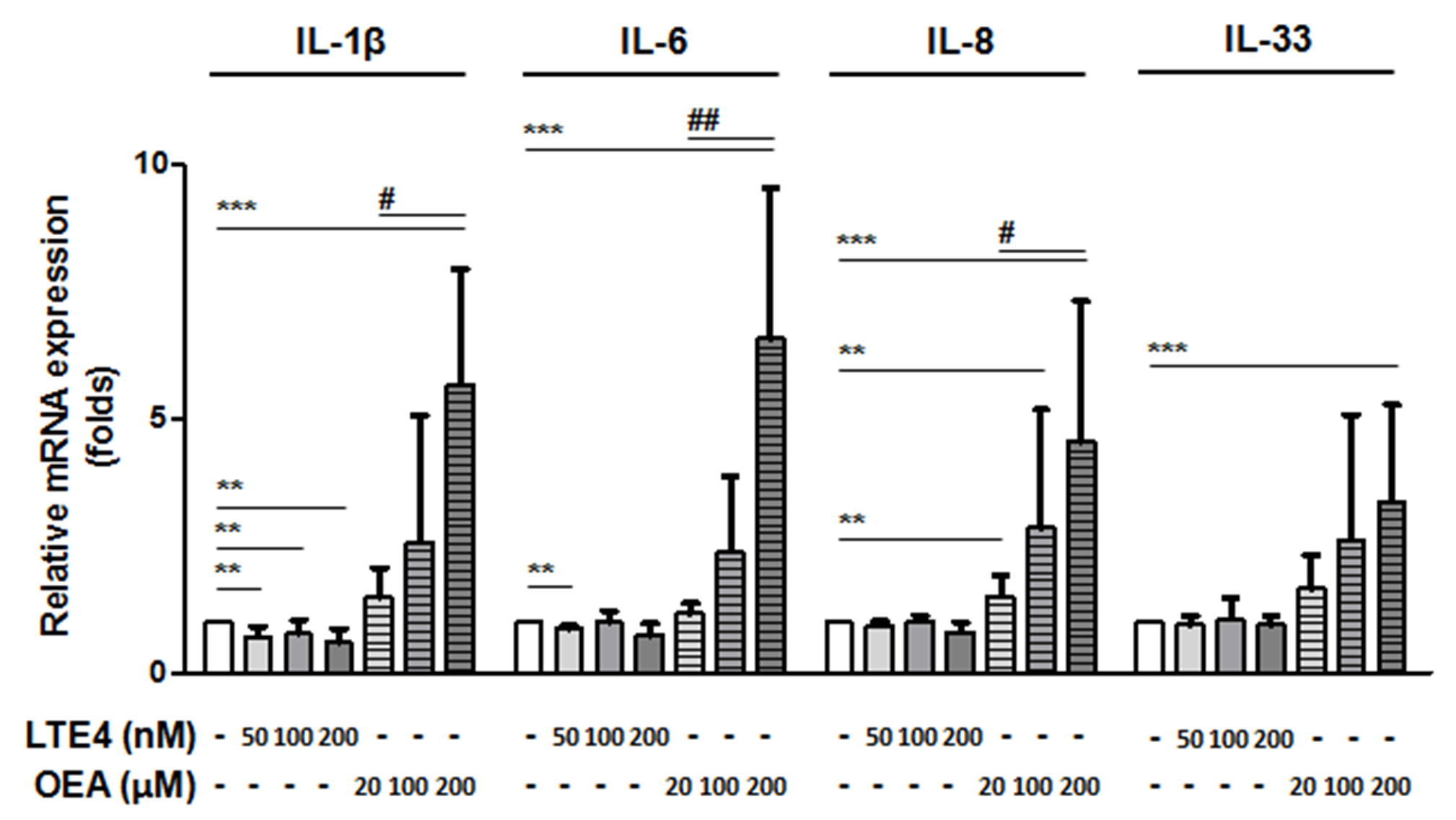
***

***Figure S3***


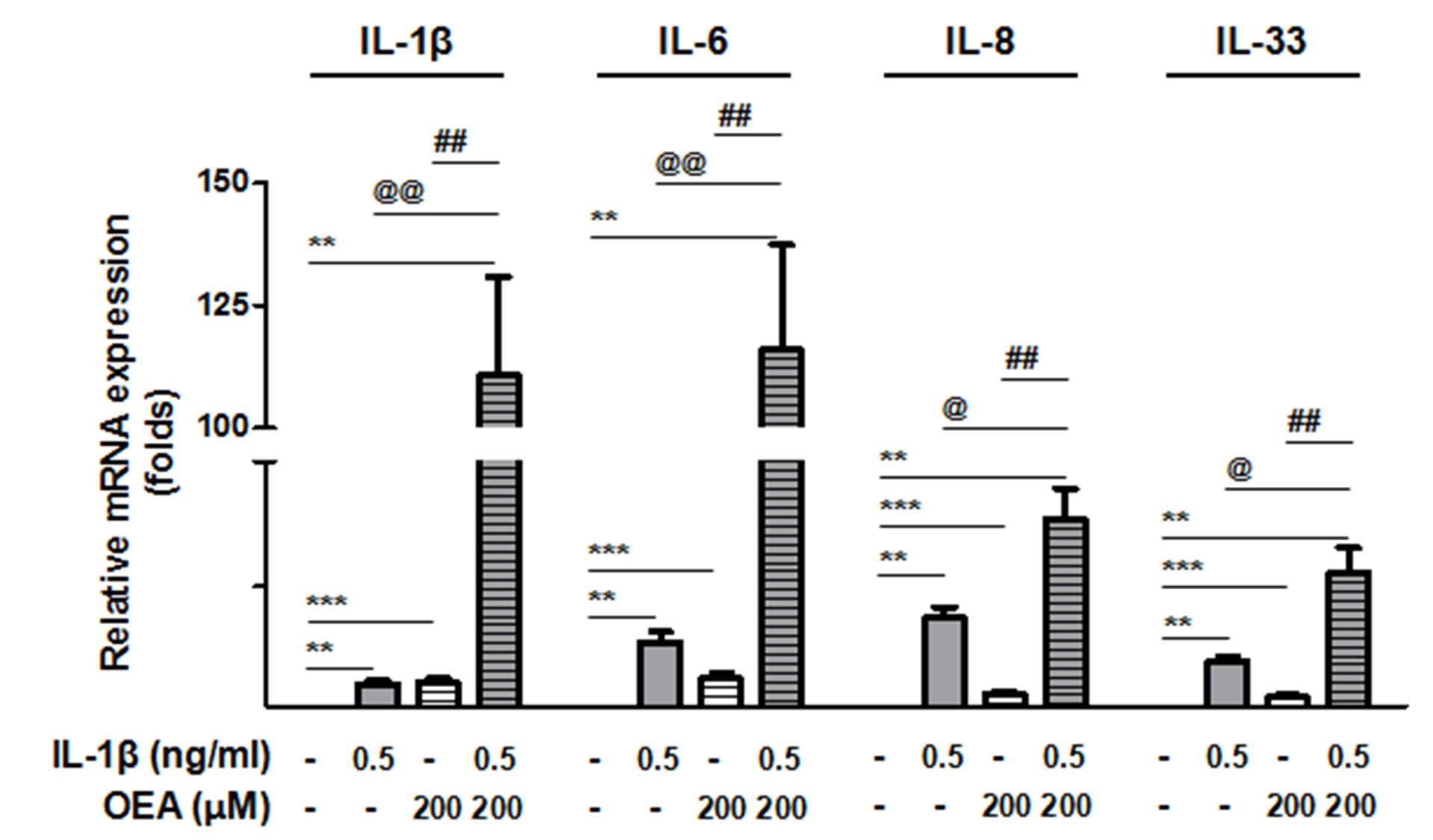


***Figure S4***

***
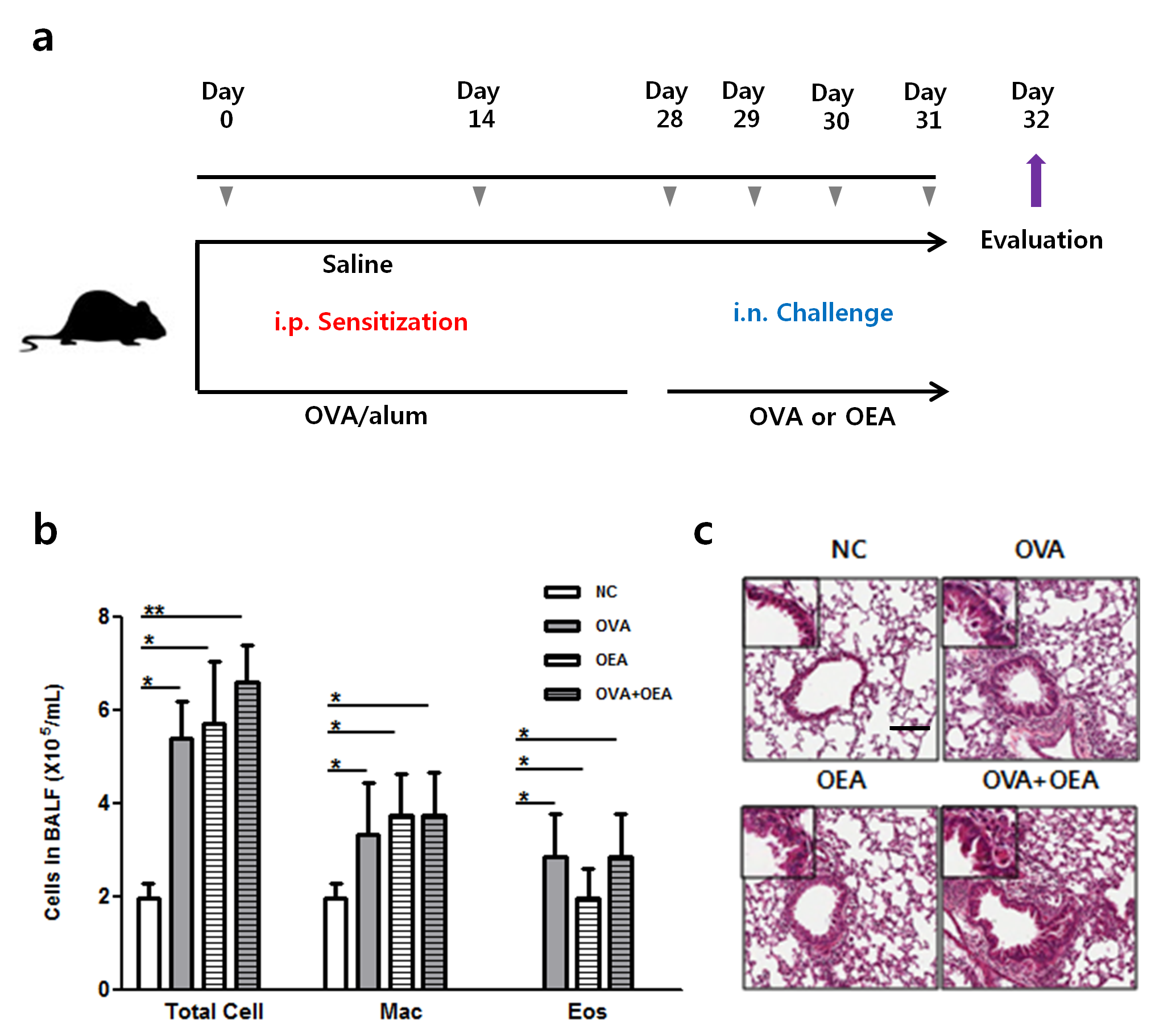
***

***Figure S5***

***
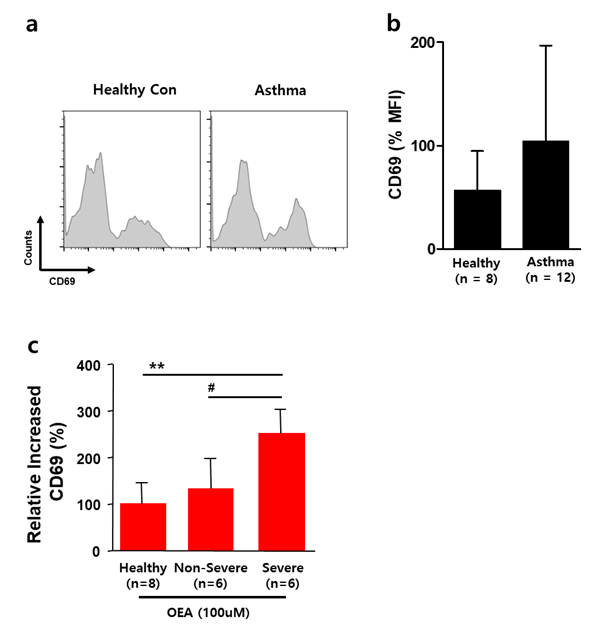
***
